# Laser and Skin‐Care Synergy: A Post‐Laser Application of Novel Adaptogenic Moisturizing Serum for Improving Healing and Cosmesis on the Face

**DOI:** 10.1111/jocd.16668

**Published:** 2024-11-06

**Authors:** Marianna Blyumin‐Karasik, Jessica Colon, Sylvie Nguyen, Jordan Rosen

**Affiliations:** ^1^ Precision Skin & Body Institute Davie Florida USA; ^2^ Nova Southeastern University Dr. Kiran C. Patel College of Osteopathic Medicine Davie Florida USA

**Keywords:** aesthetic, cosmeceuticals, healing, homeostasis, laser treatment, moisturizing serum, photoaging, skin adaptogens, skin quality, topical adaptogen

## Abstract

**Background:**

Laser procedures are often used for global improvement in skin quality. The state of the skin is under stress after laser treatment, and it is beneficial to utilize topical agents to assist with optimal healing and cosmetic outcomes. Currently, such post‐laser‐cosmeceutical synergies are being investigated. Skin adaptogens are bioactive ingredients that enhance the skin's resistance and adaptation to stress.

**Aim:**

Investigate the use of an adaptogenic moisturizing serum (AMS) in synergy with post‐laser treatment recovery.

**Patient/Methods:**

Eight patients underwent long‐pulsed 755‐nm alexandrite or 1064‐nm picosecond laser treatments on the face, targeting photoaging and pigmentation. After the laser treatment, during their healing process, they utilized a novel AMS twice daily to assist with the post‐laser recovery. A blinded expert grader evaluated before and after facial images of each patient using a modified Griffiths photodamage photonumeric scale.

**Results:**

The findings revealed that the AMS was well‐tolerated with no reported adverse events, and elicited aesthetically pleasing results. There was a significant decrease in photodamage scores following the treatment (*p* = 0.0078) with a median reduction of 3.5. Patients had improvement in skin photodamage, pigmentation, and skin radiance in clinic and photography after a combination of laser and AMS serum treatments.

**Conclusion:**

Utilizing the adaptogen moisturizing serum skin care regimen after the laser treatments likely assisted in the skin's healing process. As a result, we propose that there is a potential benefit of combining certain laser treatments and an adaptogen‐based skin care regimen for better aesthetic outcomes and skin recuperation post‐procedure.

## 1 Introduction

Treatments for minimally and noninvasive facial aesthetic procedures are currently in significant demand [1]. A survey conducted by the American Society of Dermatologic Surgery in 2019 revealed that approximately 70% of 3465 participants were contemplating a cosmetic procedure in order to enhance their confidence and maintain their youthful appearance [2]. There are many non‐invasive modalities that can be utilized in dermatology to improve skin quality by targeting photoaging and pigmentation. Some of these non‐invasive modalities include laser resurfacing, microdermabrasion, and chemical peels [3]. Lasers are important tools for aesthetic skin quality improvement [4]. Long‐pulsed lasers and short‐pulsed lasers can be used to target pigmentation and photodamage to improve skin tone [5, 6].

The alexandrite (Alex) laser utilizes an alexandrite crystal to produce a long‐pulsed wavelength of 755 nm in the infrared spectrum. Alex lasers are used to treat vascular lesions, pigmented lesions, and hair removal [5]. The wavelength and pulse duration of the Alex laser allow the laser to penetrate to the appropriate depth to absorb melanin in the skin. Studies have demonstrated the effectiveness of the Alex laser in clearing solar lentigines [7, 8].

Picosecond (Pico) lasers were developed to transmit high‐frequency light energy within a sub‐nanosecond [9]. Like the Alex laser, the picosecond laser target melanin, yet reduces nonspecific photothermal damage via more specific shockwave energy. Both the Alex and Pico lasers have been successful in treating hyperpigmentation [9].

The goal of cosmetic laser treatments is to induce a controlled skin injury which results in a wound healing process that causes structural rejuvenation and enhanced texture and tone [10]. However, the laser procedure results in temporary stress and vulnerability in the skin post‐procedure. As a result, topical agents have been investigated during laser recovery to improve cosmetic results, hasten wound healing, and minimize adverse effects. At this time, there is a need for optimal peri‐laser skin regimen [10, 11].

Topical adaptogens are generally botanical actives that improve skin resiliency and skin quality [12, 13]. Adaptogens interact with the body's natural response to environmental aggressors to enhance protective benefits and maintenance of homeostasis [13]. Evidence has shown that combining skin adaptogens provides synergistic advantages: enhancing resistance to stress, diminishing inflammation, improving skin repair physiology, and delaying premature aging [13]. Hence, they are helpful conductors to orchestrate the skin's return to a healthier state of equilibrium after the post‐procedural injured phase, such as after a laser treatment.

### 1.1 A New Adaptogenic Moisturizing Serum (AMS)

A novel serum, AMS, containing a combination of active adaptogens such as Curcumin, Gotu Kola, Cordyceps, Resveratrol, etc., was developed to help with skin resilience and overall skin quality. Furthermore, the serum contains moisturizing ingredients such as Hyaluronic Acid, Ceramides, Squalane, Glycerin, etc. In this case series, eight patients received laser therapies and then applied AMS after the laser treatment. Their healing and cosmetic outcomes are in this report.

Along with new moisturizer application, the patients were guided to follow standard post‐laser protocol including, minimal ultraviolet light exposure, regular use of mineral‐based sunscreen, gentle cleanser and makeup removal, and focal use of vaseline‐based emollient as needed during their post‐laser healing process.

## 2 Materials and Methods

**FIGURE 1:**
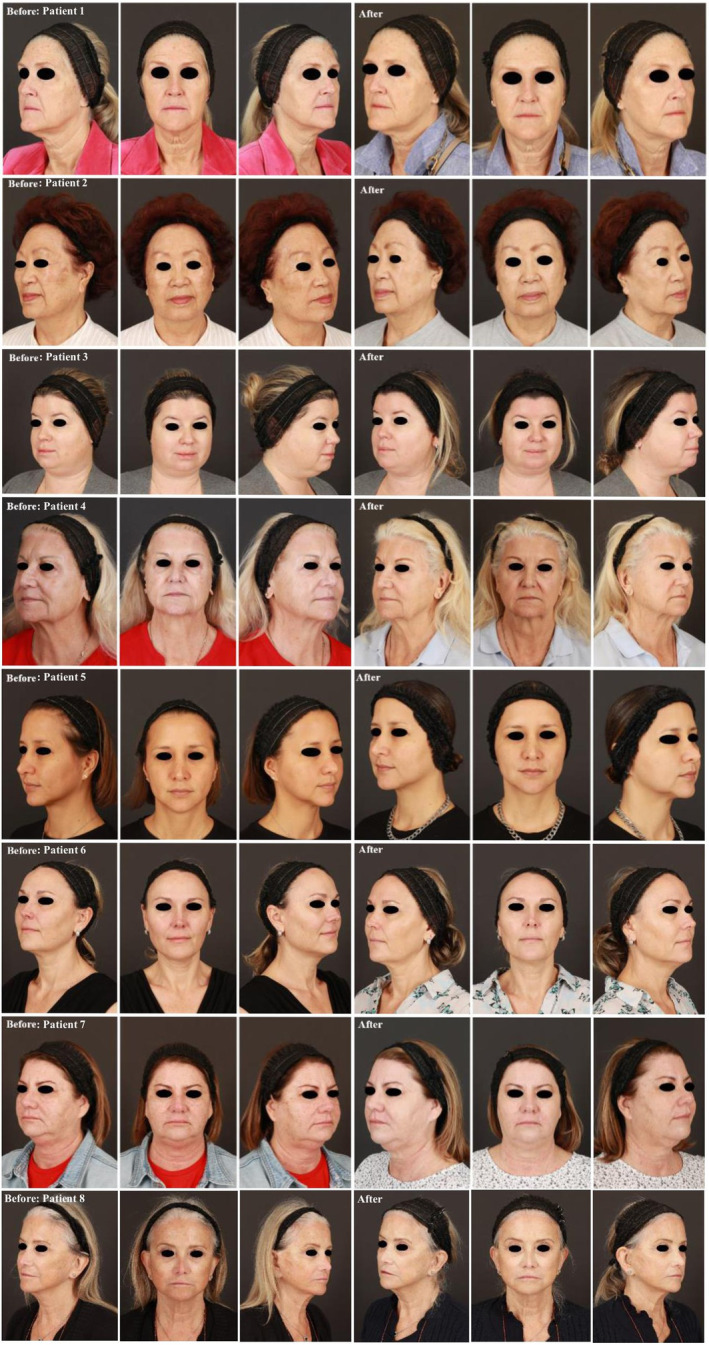
Pre/post laser and AMS treatment photos.

We present a series of eight cases where a combination of laser and post‐laser AMS skin‐care therapy were used in synergy to treat pigmentation and photodamaged skin. The laser treatments utilized were Alex (GentleMaxPro, Candela) and Pico (Picoway, Candela) and were operated by a board‐certified dermatologist in a dermatology clinic. The laser sessions were followed by AMS applied by patients twice daily starting on the night after the first laser treatment and continuing until the follow‐up. The patients' laser treatments were 6 weeks apart followed by a follow‐up visit 6 weeks after their last laser treatment. At this visit, post‐laser photography (Figure 1) and a brief assessment of the tolerability and cosmetic benefits of AMS were discussed. Information regarding each patient's treatment can be found in Table 1.

**TABLE 1:** Summary of patient treatments.

Patient	Age	Race	Gender	Fitzpatrick skin types	Laser	Treatment	AMS used twice daily	Participant comment on AMS
1	59	Caucasian	Female	2	Alex	*First treatment*: 26 J/cm^2^ using 12‐mm spot size, 3‐ms pulse width *Second treatment*: 30 J/cm^2^ using 12‐mm spot size, 3‐ms pulse width	✓	“Enjoyed the use of AMS post‐laser because it provided a brighter appearance and smoother feel of her skin.”
2	83	Asian	Female	4	Alex and 70% glycolic acid peel	*First treatment*: 24 J/cm^2^ using 12‐mm spot size, 3‐ms pulse width *Second treatment*: 20 J/cm^2^ using 18‐mm spot size, 3‐ms pulse width	✓	“Like the use of AMS post‐laser, because it improved the look and feel of her skin.”
3	42	Caucasian	Female	2	Alex	*First treatment*: 26 J/cm^2^ using 15‐mm spot size, 3‐ms pulse width *Second treatment*: 28 J/cm^2^ using 12‐mm spot size, 3‐ms pulse width	✓	“Liking the silky texture of AMS, as well as how it improved the radiance of her skin.”
4	77	Caucasian	Female	2	Alex	*First treatment*: 26 J/cm^2^ using 12‐mm spot size, 3‐ms pulse width	✓	“While using AMS her skin felt more balanced, hydrated, and less dry.”
5	39	Hispanic	Female	3	Pico	*Five treatments monthly*: 1064‐nm Zoom, fluency range of 0.9–1.0 J/cm^2^, 7‐mm spot size, 5 Hz repetition rate, 1–2 passes	✓	“While using AMS her skin felt more moisturized and glowing.”
6	44	Caucasian	Female	2	Alex	*First treatment*: 26 J/cm^2^ using 12‐mm spot size, 3‐ms pulse width	✓	“While using AMS her skin felt and looked more healthier, refreshed, and hydrated.”
7	60	Caucasian	Female	2	Alex	*First and second treatment*: 30 J/cm^2^ using 12‐mm spot size, 3‐ms pulse width	✓	“Liking the use of AMS post‐laser, because it improved smoothness, hydration, and radiance.”
8	69	Hispanic	Female	4	Alex	*First and second treatment*: 26 J/cm(2) using 12‐mm spot size, 3‐ms pulse width *Treatment of facial veins*: 1064 nm Yag, 360 J/cm(2) using 1.5‐mm spot size, 20‐ms pulse width, 1 pass, 20/20 cooling	✓	“Liking the use of AMS post‐laser, because of its silky texture and it improved skin hydration and radiance.”

### 2.1 Clinical Efficacy

**FIGURE 2:**
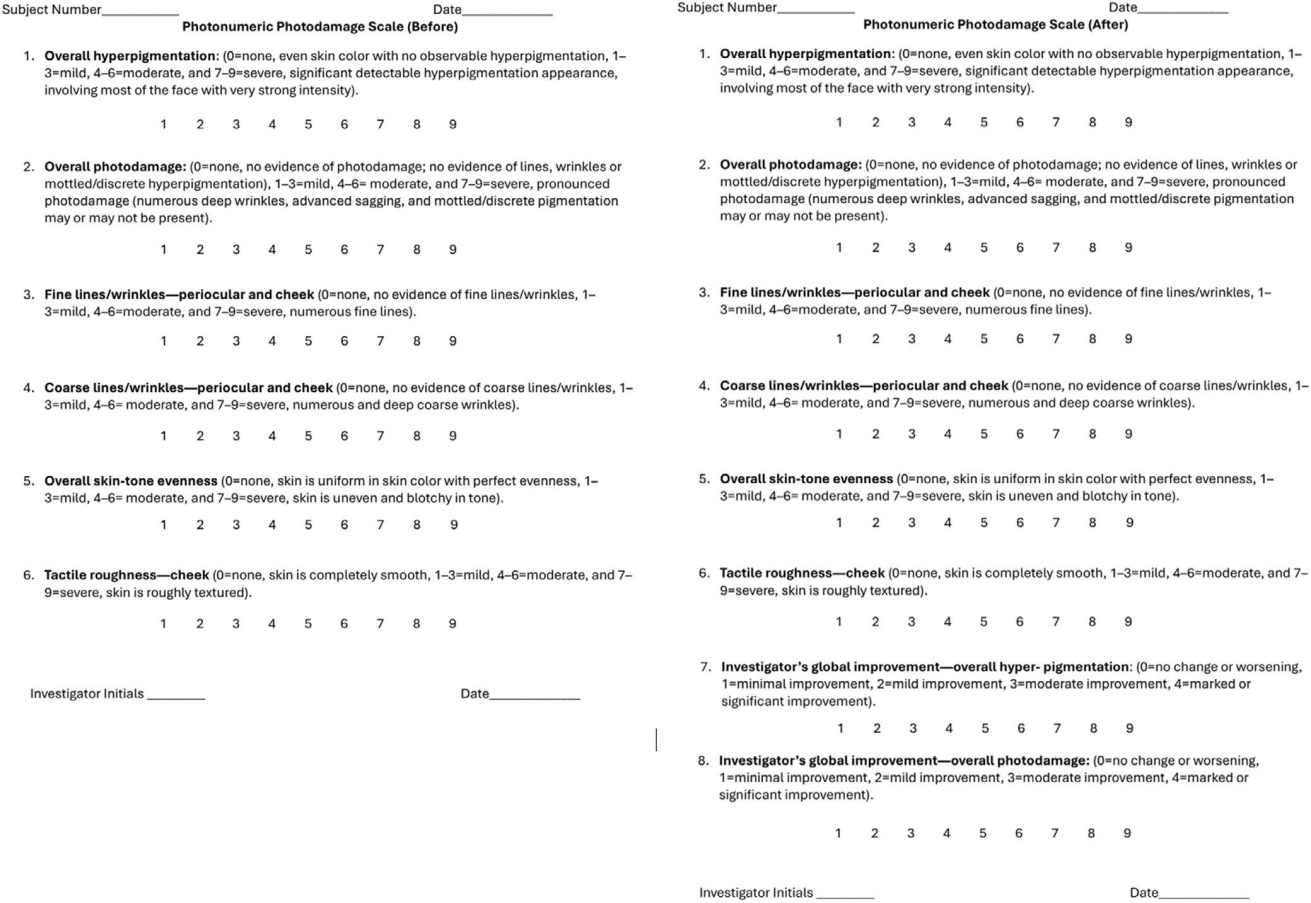
Modified griffiths photodamage photonumeric scale.

An expert grader who was blinded to the treatment protocol evaluated before and after facial images of each patient using a modified Griffiths photodamage photonumeric scale (Figure 2) [14, 15]. After photo evaluation, a Wilcoxon signed‐rank test was used to analyze the investigator's overall photodamage score from image analysis. There was a significant decrease in photodamage scores following the treatment (*p* = 0.0078) with a median reduction of 3.5.

## 3 Results

All patients reported expected skin redness, swelling, focal crusting, and exfoliation of treated pigmented lesions which lasted approximately 10 days. Patients tolerated the application of AMS well, denying any signs or symptoms of skin irritation (worsening of redness, swelling, itching, stinging, or burning sensation).

The results revealed that the post‐laser recovery was well‐tolerated and provided aesthetically pleasing results. Clinician‐reported outcomes include: 100% (8/8) of patients had improvement in skin photodamage, pigmentation, and skin radiance in clinic and photography after a combination of laser and AMS serum treatments; 100% (8/8) of patients tolerated the combination treatment without any side effects of skin irritation or pigmentation. Self‐reported patient outcomes are: 87% (7/8) participants reported skin redness and swelling and focal crusting and exfoliation of treated pigmented lesions which lasted approximately 10 days; 100% (8/8) of participants tolerated the application of AMS well and denied any signs or symptoms of irritation; 100% (8/8) participants reported positive cosmetic evaluation of AMS, commenting on its benefits of skin hydration, smoothness, and radiance as well as electing to use the AMS after procedural healing as their regular skincare routine serum.

## 4 Discussion

Laser treatments are often used for aesthetic enhancement [4] and skin care is important for improving the quality of the skin [13]. By combining two modalities, there is amplification of ideal synergy for healing and cosmetic outcomes. At this time, there is significant research underway to identify the ideal amalgamation of priming, laser‐delivery, and post‐procedural skin products to optimize the results of these aesthetic procedures [10, 11, 16].

Cosmaceuticals with adaptogens can support reactive skin, such as post‐procedural laser treatment, by modulating microbes, inflammation, hydration, and oxidative stress while inducing repair mechanisms to enhance skin healing and restore it back to health. Furthermore, some adaptogens have the capacity to repair skin healing and enhance skin quality: texture, tone, and tightness, therefore enhancing a more youthful appearance and complementing laser results [13]. Liu et al., 2023 identified 109 plant adaptogens in the literature that have demonstrated anti‐aging, anti‐photoaging, anti‐bacterial, anti‐inflammatory, and lightening capabilities in the skin [17]. Some of these adaptogens include curcumin which has anti‐microbial and anti‐aging effects, resveratrol which has antioxidant capacity, gotu kola which possesses anti‐inflammatory and wound healing benefits, and cordyceps which hydrates and reinforces the skin barrier [17, 18]. These and other beneficial skin adaptogens can be found in the AMS.

Comparison of before and after photography demonstrated improvement of pigmentation and photodamage based on blinded modified Griffiths photodamage photonumeric scale after synergistic laser and AMS skin‐care therapy (Figure 1). Utilizing the AMS skin care regimen after the laser treatments likely assisted in the skin's healing process and was well‐tolerated by participants, based on patients' self‐reporting. Another notable patient's self‐reported finding is that AMS was cosmetically pleasing, and they integrated using it as part of their regular skin care regimen, after follow‐up. As a result, we propose that there is a potential benefit of combining certain laser treatments and an AMS skin care regimen for better aesthetic outcomes.

Potential limitations of this analysis include the small sample size, its retrospective design, and reliance on patients' subjective self‐reporting evaluations. The lack of a control group makes it unclear what the clinical course of these patients would be on laser and/or AMS alone. A future split‐face design to assess the difference between laser monotherapy versus concurrent AMS and laser treatment, along with objective skin quality investigator evaluations will be helpful in this regard. Nonetheless, in this brief study, the results show that AMS was well‐tolerated and may enhance skin healing and tone when used after laser. Long‐duration, prospective controlled studies are needed to further characterize the impact of AMS as a post‐procedural cosmetic topical agent.

## Author Contributions

Marianna Blyumin‐Karasik and Jessica Colon contributed to the drafting and editing of the manuscript. Sylvie Nguyen contributed to the data analysis and Jordan Rosen performed the blinded photo evaluation.

## Ethics Statement

We would like to extend gratitude to the patients participating in the study, who were treated in the most ethical way in accordance with Good Clinical Practices.

## Consent

All the patients participating in the study agreed and signed the photo release consent for their photography for this publication.

## Conflicts of Interest

Marianna Blyumin‐Karasik, MD is a founder and CEO of Stamina Beauty, LLC, manufacturer of AMS.

## Data Availability

The data that support the findings of this study are available from the corresponding author upon reasonable request.
